# Changes in circulating bile acid levels during cold exposure are associated with brown adipose tissue in humans: *a secondary analysis from the ACTIBATE study*

**DOI:** 10.1007/s13105-026-01155-5

**Published:** 2026-02-28

**Authors:** Lucas Jurado-Fasoli, Borja Martinez-Tellez, Isabelle Kohler, Jonatan R. Ruiz, Francisco J. Osuna-Prieto

**Affiliations:** 1 Department of Physiology, Faculty of Medicine, Sport and Health University Research Institute (iMUDS), University of Granada, Granada, Andalucía, Spain; 2 Instituto de Investigación Biosanitaria (Ibs), Granada, 18014 Spain; 3 CIBER de Fisiopatología de la Obesidad y Nutrición (CIBEROBN), Instituto de Salud Carlos III, Madrid, Spain; 4 Department of Nursing, Physiotherapy and Medicine and SPORT Research Group, CIBIS Research Center, University of Almería, Almería, 04120 Spain; 5 Biomedical Research Unit, Torrecárdenas University Hospital, Almería, 04009 Spain; 6 Department of Medicine, Division of Endocrinology and Einthoven Laboratory for Experimental Vascular Medicine, Leiden University Medical Center, Leiden, The Netherlands; 7 Division of BioAnalytical Chemistry, Department of Chemistry and Pharmaceutical Sciences, Vrije Universiteit Amsterdam, Amsterdam, 1081 HV The Netherlands; 8 Center for Analytical Sciences Amsterdam, Amsterdam, 1098 HX The Netherlands; 9 Department of Physical Education and Sports, Faculty of Sports Science, Sport and Health University Research Institute (iMUDS), University of Granada, Granada, 18071 Spain; 10 Hospital Universitari Joan XXIII de Tarragona, Institut d’Investigació Sanitària Pere Virgili (IISPV), Tarragona, 43005 Spain; 11 CIBER de Diabetes y Enfermedades Metabólicas Asociadas (CIBERDEM), Instituto de Salud Carlos III (ISCIII), Madrid, 28029 Spain

**Keywords:** Brown fat, Cold-induced thermogenesis, Adaptive thermogenesis, Uncoupling-protein-1 (UCP1), Cardiometabolic health, Obesity

## Abstract

Cold exposure activates brown adipose tissue (BAT), a therapeutic target to mitigate cardiometabolic diseases. Emerging evidence suggests that bile acids may influence BAT activity through metabolic and endocrine signaling pathways. Characterizing changes in circulating bile acids during cold exposure may uncover new pathways to improve metabolic health in humans. We analysed 56 young (21.7 ± 2.3 years old), sedentary adults (43 women, 77%) from the ACTIBATE cohort (NCT02365129) before the intervention in whom plasma samples were available for bile acid analyses. Eighteen plasma bile acid species were analyzed using liquid chromatography-tandem mass spectrometry before (i.e., baseline), 60, and 120 min during a personalized cooling protocol. BAT volume, activity, and radiodensity were assessed via ^18^F-fluorodeoxyglucose positron emission tomography-computed tomography scan (^18^F-FDG PET-CT) at the end of the cooling protocol. During the 2-hour cold exposure, plasma levels of total, secondary, and conjugated bile acids decreased by 24–30% (all *P* < 0.001). In normal-weight participants, most reductions remained significant after adjustment for PET-CT time, and glycolithocholic acid (GLCA) showed consistent positive associations with BAT volume and glucose uptake, as well as an inverse association with radiodensity (|r|≤0.41, adj. FDR ≤ 0.25; *R* ≤ 0.34, *P* ≤ 0.038 in adjusted regression models). In participants with overweight or obesity, most bile-acid changes lost statistical significance after adjustment for PET-CT time, although lithocholic acid and its sulfated derivative remained positively correlated with BAT radiodensity (|r|≤0.53; adj. FDR ≤ 0.25). Acute cold exposure is associated with changes in circulating bile acids in humans, with patterns differing by body-weight status. In normal-weight individuals, GLCA displayed consistent associations with BAT volume and glucose uptake, suggesting a possible link between bile acid and BAT activation. Further studies are needed to confirm these observations and to clarify the physiological relevance of bile acid signaling in human thermogenic adaptation.

**Supplementary Information:**

The online version contains supplementary material available at 10.1007/s13105-026-01155-5.

## Introduction

Cardiometabolic diseases, including obesity, type 2 diabetes, and cardiovascular disorders, represent a significant global health challenge [1]. The increasing prevalence of these conditions underscores the need for novel preventive and therapeutic strategies [2]. In this context, cold exposure has gained increased attention as a potential intervention strategy [3] since it activates brown adipose tissue (BAT). BAT is a specialized adipose tissue that dissipates energy as heat and is regarded as a promising therapeutic target due to its capacity to mitigate cardiometabolic diseases [4, 5]. The thermogenic capacity of BAT primarily depends on the activation of uncoupling protein 1 (UCP1), whose activation is mainly driven by sympathetic nervous system (SNS) stimulation in response to cold exposure [6]. However, UCP1-independent thermogenic mechanisms have also been reported [7]. In humans, BAT activity has been associated with improved glucose metabolism, enhanced insulin sensitivity, and increased energy expenditure [8]. Epidemiological and imaging-based studies have demonstrated that active BAT is linked to cardiometabolic health in humans [9]. Nevertheless, the molecular mechanisms regulating BAT activity in humans remain incompletely understood.

In this context, bile acids have emerged as potential activators of cold-induced BAT thermogenesis [10, 11]. Primary bile acids are synthesized from cholesterol in hepatocytes, conjugated with glycine or taurine, and secreted into the duodenum via the biliary tract [12]. In the intestine, bacterial enzymes convert primary bile acids into secondary species, thereby diversifying the circulating bile acid pool [13]. Most bile acids are recycled via the enterohepatic circulation or eliminated in feces [14, 15]. However, a small fraction enters the systemic circulation, where they act as signaling factors influencing glucose, lipid, and energy metabolism [16, 17]. These effects are primarily mediated through bile acid-activated receptors, such as the membrane Takeda G protein-coupled receptor 5 (TGR5) and the nuclear receptor farnesoid X receptor (FXR), which are expressed in key metabolic tissues such as the gut, pancreas, skeletal muscle, as well as brown adipocytes [18].

In murine models, activation of the bile acid receptor TGR5 in brown adipocytes enhances UCP1-dependent thermogenesis and improves systemic glucose and lipid metabolism [19–22]. Beyond thermogenesis, cold-induced BAT activation has also been associated with bile acid–mediated remodeling of cholesterol and lipid metabolism, indicating an inter-organ axis linking white adipose tissue and BAT, liver, and gut [23]. Translating these findings to humans, oral supplementation with the primary bile acid chenodeoxycholic acid (CDCA) has been shown to increase BAT glucose uptake and energy expenditure under thermoneutral conditions [18]. However, most previous evidence arises from chronic cold exposure studies in rodents or from pharmacological bile acid administration in humans under thermoneutral conditions, which do not capture the acute and physiological dynamics of endogenous bile acid responses to cold exposure in humans (the primary physiological stimulus of BAT activation).

To date, whether acute cold exposure reshapes the circulating bile-acid profile in humans and how such remodeling relates to BAT parameters remains unknown. Here, we investigated whether a 2-h personalized cold exposure protocol alters bile acid plasma levels and whether these changes are associated with BAT parameters, including volume, metabolic activity, and radiodensity. Characterizing these acute changes may provide novel insight into the metabolic crosstalk between the liver, gut, and BAT, helping to achieve a better understanding of how bile acid signaling supports adaptive thermogenesis and cardiometabolic health in humans.

## Methods

### Study participants

This study was conducted under the framework of the ACTIBATE (ACTivating Brown Adipose Tissue through Exercise; registered in ClinicalTrials.gov, ID: NCT02365129; registration date: 2015-02-10) randomized controlled trial [24]. The study involved 145 young sedentary adults aged 18 to 25 years recruited from Granada (Spain) using social media, local media outlets, and posters. To be eligible, participants had to be sedentary (defined as engaging in less than 20 min per day of moderate-to-vigorous physical activity on less than three days per week), non-smokers, have a stable body weight over the past three months, and not be on medication. Exclusion criteria included diagnoses of diabetes, hypertension, or other significant medical conditions that could interfere with or worsen due to exercise; pregnancy; use of medication affecting energy metabolism; or frequent exposure to cold temperatures.

### Study design

This study presents an observational analysis with a repeated-measures design nested within the baseline assessments of the ACTIBATE trial [24], adhering to the STROBE guidelines for observational studies to ensure methodological rigor and transparency. Before enrolment, all individuals provided written informed consent. Ethical approval for the study was granted by the Human Research Ethics Committee of the University of Granada (approval no. 924) and the *Servicio Andaluz de Salud*, in compliance with the Declaration of Helsinki (2013). Data collection and interventions were conducted at the Sport and Health University Research Institute and the Virgen de las Nieves University Hospital, both part of the University of Granada. The study took place over two consecutive years, with four waves of data collection per year, spanning from September 2015 to June 2016 and from September 2016 to June 2017, concluding at the end of the exercise intervention.

During baseline assessments, participants underwent a 2-h cold exposure test, and blood samples were collected to investigate the effects of cold exposure. The present analyses include only those 56 participants (*n* = 56; 43 women) for whom plasma samples were available to determine bile acid concentrations. All procedures described in the present analysis were conducted during the baseline assessments of the ACTIBATE trial, before the start of the 24-week intervention period. As this was an exploratory analysis based on pre-existing baseline data from the ACTIBATE trial, no formal a priori sample size calculation was performed.

### Cold exposure and ^18^F-fluorodeoxyglucose uptake by brown adipose tissue

Before the cold exposure test and BAT assessment, the shivering threshold of each participant was determined following an incremental cooling protocol [25]. This procedure began in a warm room (22.0–23.0 °C), where participants waited for 30–45 min before entering a mildly cold room (19.5–20.0 °C). Participants then donned a water-perfused cooling vest (Polar Products Inc., Stow, OH, USA), and the water temperature was gradually reduced until it reached 3.8 °C, or until the first signs of shivering were observed. If no shivering occurred, participants remained in the cold room for an additional 45 min with water set at 3.8 °C. Shivering was visually assessed by researchers and/or self-reported by participants. The shivering threshold was defined as the water temperature at which shivering first occurred [25].

Forty-eight to 72 h after determining the shivering threshold, participants underwent a 2-h personalized cooling procedure for the assessment of BAT parameters. Cold-exposure tests and PET-CT scans were scheduled between 08:30 and 20:30 h, depending on participant availability and facility logistics. All participants were tested after a minimum 6-hour fast, and each individual’s three blood samples (baseline, 60 min, and 120 min) were collected within the same session and under identical resting and environmental conditions. This design minimized intraindividual variability related to feeding status or circadian influences. Initially, participants rested in a warm room (22.0–24.0 °C) before being moved to a cooler room (19.5–20.0 °C). They wore a water-perfused cooling vest (Polar Products Inc., Stow, OH, USA) that covered the abdomen, chest, and supraclavicular region. The water temperature was set at 4 °C above each participant’s shivering threshold. After the first hour of cold exposure, the water temperature was increased by 1 °C to prevent shivering (i.e., 5 °C above the individual’s shivering threshold). If participants reported shivering, the water temperature was further increased by an additional 1 °C.

After 1 h of cold exposure, participants received an intravenous injection of approximately 185 MBq of ^18^F-FDG. Following 2 h of cold exposure, a static PET/CT scan (Siemens Biograph 16 PET-CT, Erlangen, Germany) was performed with participants in the supine position. The PET/CT scans were analyzed using a previously established method with the Beth Israel plug-in [26] for the FIJI software [27].

An individualized standardized uptake value (SUV) threshold (1.2/[lean body mass/body mass]) and a fixed radiodensity range (−10 to −190 Hounsfield units) were applied for BAT quantification [28–30]. The region of interest (ROI) was semi-automatically outlined from cervical vertebra 1 to approximately the mid-chest region. BAT volume and ^18^F-FDG uptake (SUVmean and SUVpeak) were quantified following BARCIST 1.0 recommendations [28]. All scans were visually inspected for ^18^F-FDG uptake in BAT-specific depots. BAT volume was calculated based on the number of pixels in the specified radiodensity range with an SUV value above the threshold. BAT activity was measured using SUVmean (the average quantity of ^18^F-FDG in these pixels) and SUVpeak (the mean value from the three highest ^18^F-FDG concentrations in three pixels within a volume of less than 1 cm³). BAT mean radiodensity was determined as the average radiodensity of voxels meeting these criteria within the ROI, spanning from the atlas to thoracic vertebra 4, excluding the oral cavity.

A one-slice ROI was also drawn in the descending aorta (reference tissue) at the height of thoracic vertebra 4, and its SUVpeak was obtained as abovementioned. An ROI of subcutaneous white adipose tissue (scWAT) was also established in the dorsocervical and triceps area. The natural calendar day on which the ^18^F-FDG-PET/CT scans were conducted was recorded, starting from day 1 (January 1 st) and continuing through day 365 or 366 (December 31 st), depending on the year.

### Blood sample collection

Before the cold exposure, an intravenous catheter was inserted into the antecubital vein, and blood samples were collected at baseline (before) and at 60 and 120 min during the cold exposure. Additional blood samples for assessing cardiometabolic risk factors were collected on a separate day between 8:00 and 11:00 a.m. after a 10-h fast. Blood was immediately centrifuged to separate serum, collected using Vacutainer^®^ SST™ II Advance tubes, and plasma, collected using Vacutainer^®^ Hemogard™ tubes containing ethylenediaminetetraacetic acid (EDTA) as an anticoagulant. All samples were aliquoted and stored at −80 °C for later analysis.

### Determination of Circulating bile acids

Bile acid plasma levels were determined using a validated liquid chromatography-tandem mass spectrometry (LC-MS/MS) method as described elsewhere [31]. The LC-MS/MS method enabled the analysis of cholic acid (CA), chenodeoxycholic acid (CDCA), glycocholic acid (GCA), glycochenodeoxycholic acid (GCDCA), taurochenodeoxycholic acid (TCDCA), deoxycholic acid (DCA), ursodeoxycholic acid (UDCA), lithocholic acid (LCA), glycodeoxycholic acid (GDCA), glycoursodeoxycholic acid (GUDCA), glycolithocholic acid (GLCA), taurodeoxycholic acid (TDCA), tauroursodeoxycholic acid (TUDCA), taurolithocholic acid (TLCA), taurolithocholic acid 3-sulfate (TLCA-3 S), lithocholic acid 3-sulfate (LCA-3 S), hyocholic acid (HCA), and taurohyodeoxycholic acid (THDCA) (Table S1). Detailed information on how the LC-MS/MS method for bile acids and circulating cardiometabolic risk factors can be found in the **Supplementary Material.**

### Anthropometric and body composition

Weight and height were measured with participants barefoot and wearing light clothing using a Seca scale and stadiometer (model 799; Electronic Column Scale, Seca, Hamburg, Germany). These measurements were used to calculate body mass index (BMI; kg/m²). Waist circumference (WC) was measured at the narrowest point of the abdomen at the end of a normal exhalation, with participants’ arms relaxed at their sides. If the minimum perimeter could not be identified, the measurement was taken just above the umbilicus in a horizontal plane. WC was measured twice using a plastic measuring tape, and the average of these two measurements was used for analysis. Lean mass, fat mass, and visceral adipose tissue (VAT) mass were assessed using dual-energy X-ray absorptiometry (DXA) with a Discovery Wi scanner (Hologic Inc., Bedford, MA, USA). The fat mass percentage was also derived from the DXA scan.

### Statistical analyses

Data normality was initially assessed using the Shapiro-Wilk test, along with visual inspections of histograms and Q-Q plots. None of the bile acids exhibited a normal distribution, so all values were log2-transformed for further analysis. Similarly, none of the cardiometabolic risk factors followed a normal distribution, prompting the log10 transformation of their values for analysis.

Repeated-measures ANOVA was used to evaluate changes in plasma bile acid levels over time during the 2-hour cooling protocol. The log2 fold changes relative to baseline were calculated for the 60- and 120-min timepoints. Repeated measures ANOVA analyses were performed using the Statistical Package for the Social Sciences v.26.0 (IBM Corporation, Chicago, IL, USA). Because circulating bile acids may exhibit diurnal variation even under fasting conditions [32, 33], we accounted for potential time-of-day effects in all analyses to ensure that any observed changes reflected cold exposure per se. The subsequent analyses examined associations between changes in bile acids and BAT parameters. We conducted Pearson partial correlation analyses between cold-induced changes in bile acids (i.e., 120-min fold changes) and BAT parameters (i.e., BAT volume, SUVmean, SUVpeak, and mean radiodensity). Accordingly, all models were adjusted for both calendar day of the baseline PET-CT acquisition (to account for seasonal variations in BAT activity [34, 35]) and PET-CT time to control for potential circadian variation in bile acid levels. We also performed multiple linear regression analyses between the levels of GLCA at the 120-min time point with log2 fold change rel. to baseline and BAT-related outcomes adjusted for calendar day of the baseline PET/CT acquisition and PET-CT time. Then, we conducted Pearson bivariate correlation analyses between cold-induced changes in bile acid levels and the ^18^F-FDG uptake of reference tissues (i.e., scWAT dorsocervical, scWAT triceps, and descending aorta) and the water temperature of the cooling vest. Pearson bivariate correlation analyses were further performed to evaluate the associations between cold-induced changes in bile acid levels (i.e., 120-min fold-change) and cardiometabolic risk factors. All Pearson bivariate correlation analyses were adjusted for multiple comparisons using the two-stage step-up Benjamini–Hochberg false discovery rate (FDR) procedure, with an FDR threshold set at 0.25 (reflecting the exploratory nature of the analysis). Correlation analyses were performed using R software version 3.6.0 (R Foundation for Statistical Computing).

All analyses were performed for all participants, as well as for normal-weight and overweight or obese participants separately. Figures were built with GraphPad Prism software v.9 (GraphPad Software, San Diego, CA, USA) and the R software version 3.6.0 (R Foundation for Statistical Computing). Statistical significance was set at *P* < 0.05.

## RESULTS

The anthropometric and metabolic characteristics of the study participants are shown in Table 1.

**Table 1:** Characteristics of the study participants

	All (*n* = 56)		Men (*n* = 13)		Women (*n* = 43)	
	Mean	SD	Mean	SD	Mean	SD
Age (years)	21.7	2.3	22.6	2.7	21.5	2.1
*Body composition*						
BMI (kg/m^2^)	24.6	5.0	28.0	6.7	23.5	3.9
Waist circumference (cm)	81.0	15.3	93.5	19.6	77.1	11.4
Lean mass (kg)	41.1	9.4	54.1	7.9	37.2	5.4
Fat mass (kg)	25.1	9.9	28.2	15.6	24.2	7.4
Fat mass (%)	36.0	7.4	30.9	10.5	37.5	5.6
VAT mass (g)	315.3	197.6	470.5	234.7	268.4	160.3
*Cardiometabolic risk factors*						
Glucose (mg/dL)	88.2	7.0	90.5	9.9	87.4	5.8
Insulin (µIU/mL)	8.8	6.9	11.9	11.9	7.9	4.2
HOMA-IR	2.0	1.9	2.9	3.4	1.7	1.0
Total cholesterol (mg/dL)	170.7	36.0	170.4	47.7	170.8	32.2
HDL-C (mg/dL)	55.1	11.7	46.2	5.2	57.8	11.8
LDL-C (mg/dL)	98.9	28.5	102.8	35.6	97.7	26.3
Triglycerides (mg/dL)	91.0	66.6	113.2	89.2	84.1	57.5
APOA1 (mg/dL)	153.9	34.0	129.4	15.3	158.8	34.7
APOB (mg/dL)	68.6	19.2	75.1	20.6	67.3	18.9
Adiponectin (mg/L)	11.8	8.6	7.3	6.0	13.1	8.9
Leptin (µg/L)	6.2	4.0	4.1	3.8	6.8	4.0
GPT (IU/L)	20.0	20.1	34.8	34.9	15.2	8.7
GGT (IU/L)	20.5	22.9	37.8	38.5	15.0	11.1
ALP (IU/L)	75.7	22.5	87.5	26.5	72.1	20.0
Creatinine (mg/dL)	0.8	0.1	0.9	0.2	0.7	0.1
Creatine kinase (U/L)	108.5	106.8	184.8	195.0	84.9	38.2
C-reactive protein (mg/L)	2.9	4.1	3.5	3.2	2.7	4.4
*Brown adipose tissue*						
BAT volume (mL)	67.0	64.6	85.4	87.7	61.4	55.9
BAT SUVmean	3.6	1.8	3.4	1.3	3.7	2.0
BAT SUVpeak	10.8	7.9	11.0	8.7	10.7	7.8
BAT radiodensity (HU)	−59.3	8.8	−60.0	9.6	−59.1	8.6

Data presented as mean and standard deviation (SD). BAT radiodensity sample size: all, *n* = 40; men, *n* = 9; women, *n* = 31

*ALP* alkaline phosphatase, *APOA1* apolipoprotein A1, *APOB* apolipoprotein B, *BAT* brown adipose tissue, *BMI* body mass index, *GGT* gamma-glutamyl transferase, *GPP* glutamic pyruvic transaminase, *HDL-C* high-density lipoprotein cholesterol, *HOMA-IR* homeostatic model assessment of insulin resistance index, *HU* Hounsfield Units, *LDL-C* low-density lipoprotein cholesterol, *SUV* standardized uptake value, *VAT* visceral adipose tissue

### Cold exposure induces a selective remodeling of circulating bile acid profiles in a body weight-dependent manner

**Fig. 1:**
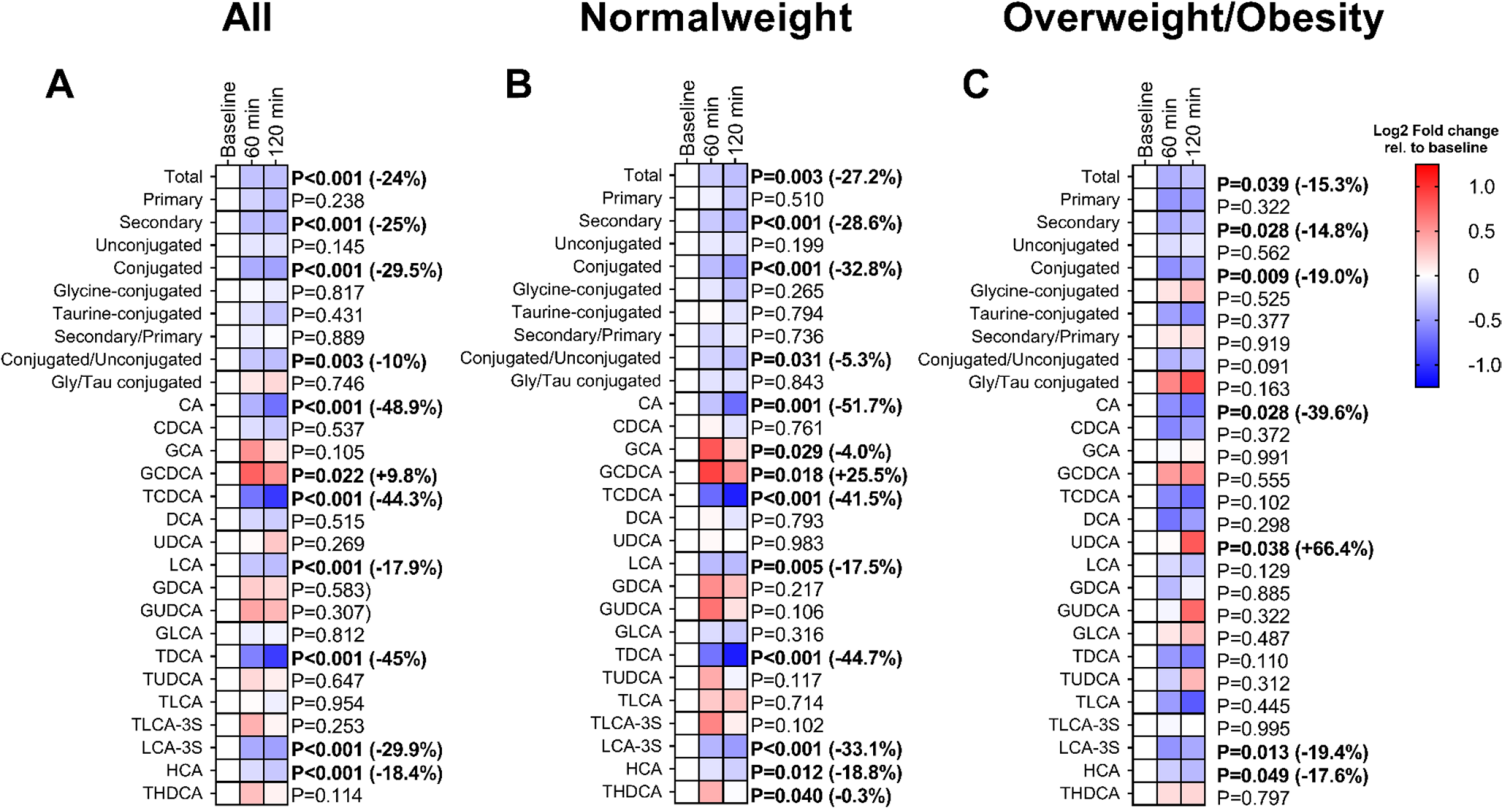
Effects of a 2-h cold exposure on plasma levels of bile acids in all participants (A; *n* = 56), normal-weight individuals (B; *n* = 19), and individuals with overweight/obesity (C; *n* = 37). The colors of the squares represent the mean log2 fold change of the peak area ratio of that time point relative to the baseline. Red color represents an increase, whereas blue represents a decrease, respectively. P values were obtained from repeated measures analyses of variance (ANOVA). Values in brackets represent the percentage of change at 120 min relative to baseline. The names, abbreviations, and respective internal standards of bile acids are detailed in Table S1

During the 2-hour cold exposure, plasma levels of total, secondary, conjugated, and conjugated/unconjugated bile acids decreased by −24%, −25%, −29.5%, and − 10%, respectively (all P *≤* 0.003; Fig. 1A). Likewise, plasma levels of CA, TCDCA, LCA, TDCA, LCA-3 S, and HCA were reduced by −48.9%, −44.3%, −17.9%, −45%, −29.9%, and − 18.4%, respectively (all *P* < 0.001; Fig. 1A). In contrast, circulating GCDCA levels increased by + 9.8% (*P* = 0.022; Fig. 1A). In both normal weight individuals and individuals with overweight/obesity (OW/OB), the plasma levels of total, secondary, and conjugated bile acids were reduced, as well as CA, LCA-3 S, and HCA (−18% to −49%, all *P* ≤ 0.049; Fig. 1B-C), whereas plasma levels of conjugated/unconjugated bile acids, TCDCA, LCA, and TDCA were reduced exclusively in normal-weight individuals (−5% to −45%, all *P* ≤ 0.031; Fig. 1B). Circulating levels of GCA, GCDCA, and THDCA increased only in individuals with normal weight (all *P* ≤ 0.04; Fig. 1B), whereas circulating UDCA levels only increased in individuals with OW/OB (*P* = 0.038; Fig. 1C). When analyses were adjusted for the exact time of PET-CT acquisition (i.e., just after the cold exposure session), the overall pattern of cold-induced bile acid remodeling remained consistent (Table S3). The main decreases in total, secondary, and conjugated bile acids, as well as in CA, TCDCA, TDCA, LCA-3 S, and HCA, remained significant after adjustment, particularly in normal-weight individuals. However, most of the previously significant changes in the OW/OB subgroup lost statistical significance after time adjustment (Table S3).

### Associations between GLCA changes during cold exposure are linked to BAT volume, activity, and radiodensity in normal-weight individuals, but not in those with overweight/obesity

**Fig. 2:**
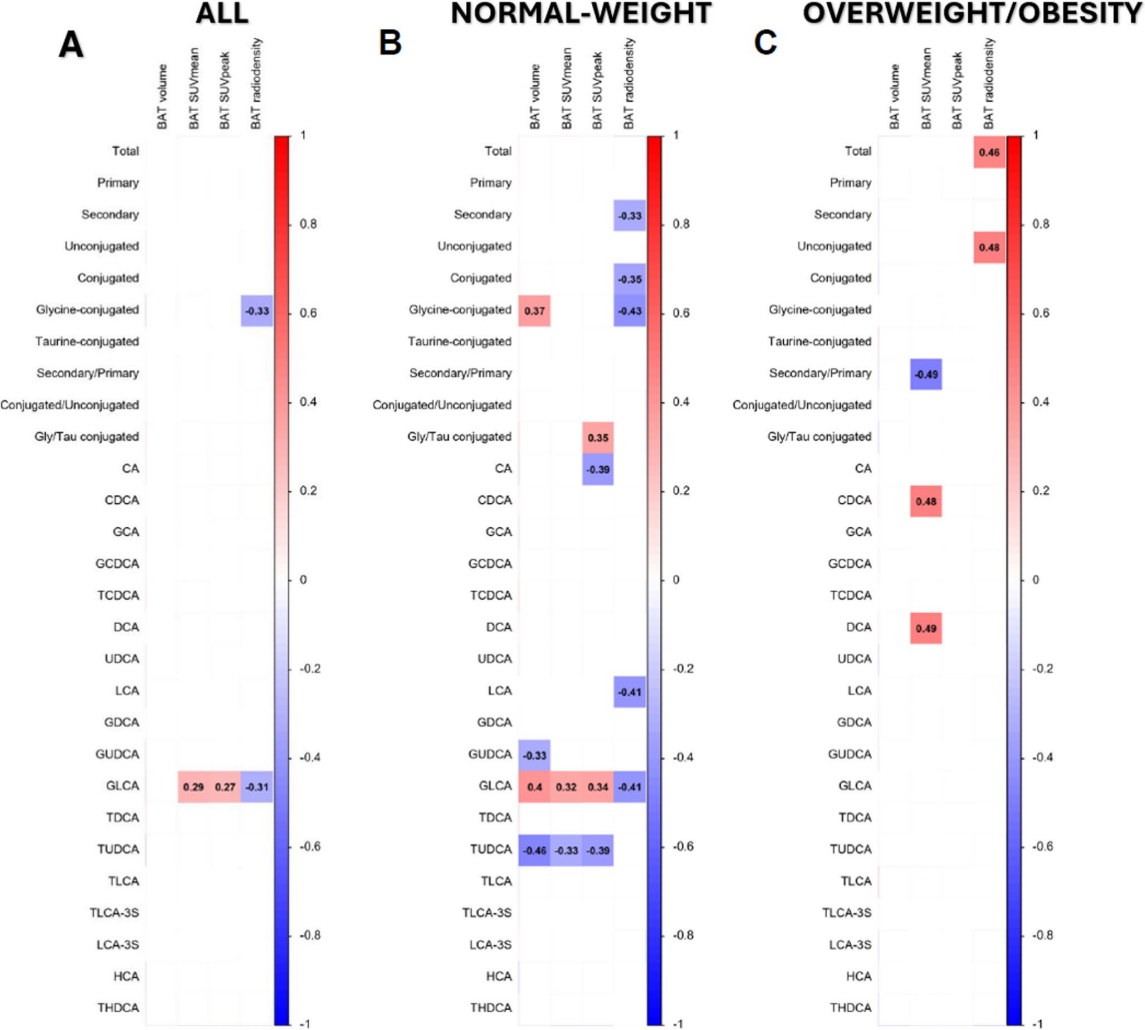
Association between cold-induced changes in the plasma levels of bile acids and brown adipose tissue-related outcomes in all participants (A; *n* = 56)>, normal-weight individuals (B; *n* = 19), and individuals with overweight/obesity (C; *n* = 37). Pearson partial correlation analyses between the log2 120-min fold change relative to baseline and BAT-related outcomes adjusted for calendar day of the baseline PET/CT acquisition. Every box represents a significant correlation coefficient (all *p* < 0.05 after FDR correction), whereas empty spaces represent no significant correlations. Red and blue boxes indicate positive and negative correlations, respectively. *Abbreviations*: BAT, brown adipose tissue; SUV, standardized uptake value. The names and abbreviations of bile acids are detailed in Table S1

In the full sample, changes in glycine-conjugated bile acid levels were inversely correlated with BAT radiodensity (*r*=−0.33; adj. FDR ≤ 0.25; Fig. 2A). GLCA changes were positively associated with BAT activity (i.e., SUVmean and SUVpeak; *r* ≥ 0.27; adjusted FDR ≤ 0.25; Fig. 2A**)** and inversely with radiodensity **(***r*=−0.31; adjusted FDR ≤ 0.25; Fig. 2A**).** Multiple linear regression adjusted for PET-CT time and calendar day showed that 120-min GLCA log₂ fold-change predicted BAT SUVmean and SUVpeak, and BAT volume (β = 0.252; R²=0.350; *P* = 0.038; Fig S2A) along with correlations of similar direction (|r|=0.32–0.41; adj. FDR ≤ 0.25; Fig S1A). In normal-weight participants, multiple linear regression adjusted for PET-CT time and calendar day showed that 120-min GLCA log₂ fold-change predicted BAT volume, BAT SUVmean, and SUVpeak (**R²**=0.19–0.35; *P* ≤ 0.038; Fig S2B) in line with correlations of similar direction (|r|=0.32–0.41; adj. FDR ≤ 0.25; Fig S1B). In participants with OW/OB, total and unconjugated bile acids were positively correlated with BAT radiodensity, whereas CDCA and DCA were positively associated with BAT SUVmean (all |r|≥0.46; adj. FDR ≤ 0.25; Fig. 2C). After adjustment for PET-CT time, associations between total, secondary, and unconjugated bile acids with BAT radiodensity persisted, while LCA and its sulfated derivative LCA-3 S emerged as the bile-acid species related to radiodensity (|r|≥0.46; adj. FDR ≤ 0.25; Fig. S1C).

Changes in plasma levels of glycine-conjugated bile acids and GLCA were not associated with the activity of reference tissues, including dorsocervical and triceps scWAT or descending aorta SUVpeak, or with the cooling vest temperature (all *P* > 0.05; Fig. S3).

## Discussion

Our study shows that a 2-hour cold exposure is associated with reduced circulating levels of total and secondary bile acids in humans. These associations remained robust after adjusting for the time of day of exposure, particularly among normal-weight individuals, whereas most of the relationships observed in the OW/OB group disappeared after adjustment. In normal-weight, but not in OW/OB individuals, GLCA exhibited positive associations with BAT volume and glucose uptake, indicating a potential relationship with BAT activity. While causal relationships cannot be established, our findings indicate that cold exposure alters circulating bile acid profiles in a manner that may relate to BAT function. Most of the bile acid species decreased during cold exposure (i.e., CA, TCDCA, LCA, TDCA, LCA-3 S, and HCA) are dependent on hepatic secretion, microbial transformation, or intestinal reabsorption [36, 37], suggesting that cold may transiently influence enterohepatic recirculation. A plausible explanation involves sympathetic nervous system activation, which promotes norepinephrine release and α-adrenergic splanchnic vasoconstriction, potentially reducing gastrointestinal perfusion and thereby limiting intestinal bile acid transport or reabsorption [38, 39]. This reduction in perfusion could, in turn, restrict the function of intestinal bile acid transporters, such as apical sodium-dependent bile acid transporter (ASBT) and the organic solute transporter alpha-beta (OSTα/β) [39, 40], thereby reducing intestinal reabsorption and contributing to the transient decline in circulating bile acid concentrations. For instance, CA recirculation largely depends on ASBT-mediated uptake, whereas secondary and conjugated species such as LCA-3 S and HCA rely on both microbial conversion and OSTα/β-mediated export [41]. In parallel, cold-induced sympathetic activation has been shown to repress hepatic bile acid synthesis through downregulation of cholesterol 7α-hydroxylase (CYP7A1), the rate-limiting enzyme of the classical biosynthetic pathway. This regulatory effect likely involves adrenergic signaling cascades that engage nuclear receptors such as FXR and the small heterodimer partner (SHP), which coordinate the transcriptional feedback loop controlling bile acid homeostasis [23, 42]. Moreover, elevations in circulating fibroblast growth factor 21 (FGF21) - a cold-inducible hormone that correlates with BAT volume and activity [43, 44] - may further repress bile acid synthesis, acting in concert with adrenergic and FXR-SHP–mediated regulatory pathways.

The mechanisms underlying the observed reductions in circulating bile acids remain speculative and likely involve additional physiological pathways, including microbiota-dependent bile acid biotransformation. Cold-induced reductions in gut perfusion could transiently attenuate microbial activity, thereby limiting the conversion of primary into secondary bile acids [45]. Some preclinical studies have reported increases in circulating bile acids during chronic cold exposure [23], but these protocols differ substantially from our acute human setting and induce adaptive compensations such as enhanced bile acid synthesis. Therefore, differences in temporal scales, experimental design, and physiological context likely account for the divergent outcomes. Moreover, thermogenic responses to cold differ substantially between rodents and humans, with outcomes strongly influenced by exposure duration and physiological context [39, 46]. Consequently, rather than contradictory, the human and rodent findings likely reflect different aspects of bile acid regulation in response to cold exposure.

Interestingly, we observed a body weight-dependent remodeling of circulating bile acid levels during to cold exposure. Although circulating bile acids may follow mild diurnal patterns even under fasting conditions, all analyses were adjusted for PET-CT acquisition time to account for this effect. In normal-weight individuals, most associations remained significant after this adjustment, in line with reports indicating that conjugated bile acids remain stable during prolonged fasting [47]. This supports the interpretation that cold exposure itself, rather than diurnal fluctuation, drives the short-term remodeling of circulating bile acid profiles. These reductions may transiently modulate bile acid–mediated signaling pathways, contributing to adjustments in energy metabolism during thermogenic activation. This interpretation is consistent with the documented cold-induced upregulation of FGF21 and peroxisome proliferator-activated receptor gamma coactivator 1-alpha (PGC-1α), which together promote energy mobilization while suppressing bile acid synthesis [23, 48]. The resulting bile acid profile among normal-weight individuals may thus reflect a metabolically efficient adaptation to thermogenic stress, supporting systemic homeostasis [49]. On the contrary, in individuals with OW/OB, most cold-induced changes in circulating bile acids lost statistical significance after adjustment for the time of exposure. Given that obesity is often associated with alterations in gut microbiota composition and disrupted hepatic–microbial communication [50–52], these factors could contribute to a blunted bile acid response to cold. However, the limited magnitude and consistency of these changes preclude firm conclusions and warrant further investigation under controlled temporal conditions.

In mice, bile acids such as CDCA activate TGR5 in BAT, enhancing UCP1-mediated thermogenesis independently of adrenergic input [22]. In humans, short-term oral CDCA supplementation increases BAT has been reported to increase BAT activity under thermoneutral conditions [18], supporting the idea that bile acid–mediated signaling can influence BAT metabolism beyond sympathetic control. In our study, glycine-conjugated bile acids, particularly GLCA, showed the most consistent associations with BAT parameters in normal-weight individuals, being positively related to BAT volume and glucose uptake and inversely related to radiodensity. While causality cannot be inferred, associations with BAT volume and glucose uptake are more physiologically meaningful, as these PET-derived metrics directly reflect tissue glucose uptake during cold activation [53]. In contrast, radiodensity reflects tissue composition at a single time point and is influenced by factors such as lipid content, perfusion, or measurement timing [54–57]. Therefore, the inverse association between GLCA and radiodensity likely reflects interindividual variability in BAT activation or timing of lipid mobilization rather than a direct effect of GLCA on BAT composition. Although causality remains to be demonstrated in interventional or tracer studies, the relationship between GLCA and BAT supports a potential metabolic crosstalk during thermogenic activation, deserving deeper investigation.

Previous studies indicate that, although the prevalence of BAT is reduced in obesity, its capacity for thermogenic activation can be preserved [58]. This may partly explain the persistence of correlations in the OW/OB group, where LCA and its sulfated derivative LCA-3 S remained positively associated with BAT radiodensity after PET-CT time adjustment. Because acute BAT activation during cold exposure promotes lipid mobilization, increased perfusion, and tissue hydration, these shifts typically lead to increased radiodensity values [56, 57]. LCA is a potent endogenous agonist of the G protein–coupled bile acid receptor TGR5, which promotes cAMP-dependent thermogenic signaling and UCP1 expression in brown adipocytes in experimental models [59, 60]. In contrast, its sulfated form, LCA-3 S, displays negligible receptor activity and primarily reflects hepatic detoxification processes [61, 62]. Therefore, these correlations may not necessarily indicate direct TGR5-mediated activation of BAT but rather parallel physiological adaptations (e.g., such as hepatic–intestinal bile acid metabolism or sympathetic regulation) that could influence BAT lipid composition and radiodensity during cold exposure.

## Limitations

Our study provides a comprehensive characterization of the acute bile acid response to cold exposure in a well-phenotyped cohort of humans. First, although we adjusted for the exact time of PET-CT acquisition to account for potential circadian variation in circulating bile acids, this statistical correction cannot fully replace an experimental control condition. The ideal design would include a time-matched thermoneutral session or crossover protocol to isolate the specific contribution of cold exposure from endogenous circadian or fasting-related fluctuations, and, therefore, our causal inferences should be considered tentative. Second, the use of a static ¹⁸F-FDG PET/CT limits the ability to capture real-time changes in circulating bile acids and BAT parameters [63]. Finally, although ¹⁸F-FDG PET is widely used to assess BAT activity, FDG uptake primarily reflects general glucose uptake rather than tissue-specific metabolic functions or thermogenesis [55]. Therefore, future studies should combine dynamic PET-CT and alternative PET tracers to better characterize the impact of cold-induced bile acids on BAT metabolism in humans [64].

## Conclusion

In summary, our results indicate that acute cold exposure is associated with changes in circulating bile acid profiles with patterns that differ according to body weight status in humans. Among these, GLCA displayed the most consistent associations with BAT volume and glucose uptake in normal-weight individuals, suggesting a possible link between bile acid dynamics and BAT activation. Further studies are needed to confirm these observations and to clarify the physiological relevance of bile acid signaling in human thermogenic adaptation.

## Supplementary Information

Below is the link to the electronic supplementary material.

Supplementary Material 1

## Data Availability

All relevant data are included in the article; further enquiries can be directed to the corresponding authors.

## Declarations

### Database and data handling procedures

The study adheres to a data management plan compliant with the regulations of the University of Granada, ensuring full data protection throughout the project. All data are processed in accordance with EU Regulation 2016/679 (applying measures such as pseudonymization and strict access control) and in line with Spanish national legislation (Seventeenth Additional Provision, processing of health data) established in Organic Law 3/2018 of December 5th, on the Protection of Personal Data and the Guarantee of Digital Rights. Research data and materials are securely stored following institutional guidelines and archived in a centralized biobank. Additionally, our data management strategy is aligned with the FAIR principles promoted by the Horizon 2020 framework, aiming to make data Findable, Accessible, Interoperable, and Reusable for the scientific community.
