# Supplementary material for: Changes in circulating bile acid levels during cold exposure are associated with brown adipose tissue in humans: a secondary analysis from the ACTIBATE study
Source: J Physiol Biochem. 2026 Feb 28;82(1):18. doi: 10.1007/s13105-026-01155-5 (PMC12950022; doi:10.1007/s13105-026-01155-5)
Supplement: Supplementary file 1 — Supplementary Material 1 [file 13105_2026_1155_MOESM1_ESM.docx]

**SUPPLEMENTAL MATERIAL**

***Liquid chromatography-tandem mass spectrometry***

Bile acids were first extracted using liquid-liquid extraction, following a previously published method with minor adaptations, with a starting volume of 50 µL of plasma [1, 2]. The extracted samples were analyzed using a Shimadzu LC system (Shimadzu Corporation, Kyoto, Japan) hyphenated to a SCIEX QTRAP 7500+ mass spectrometer (SCIEX, Framingham, MA, USA). Chromatographic separation was carried out on a BEH C18 column (50 mm × 2.1 mm, 1.7 μm) from Waters Technologies (Milford, MA, USA), maintained at 40°C. The mobile phase consisted of 0.1% acetic acid in water (v/v; eluent A), acetonitrile/0.1% acetic acid in methanol (90:10, v/v; eluent B), and 0.1% acetic acid in isopropanol (v/v; eluent C). Separation was performed at a flow rate of 0.7 mL/min using the following gradient: starting with 20% B and 1% C, the gradient changed to 85% B from 0.75 to 14 min and 15% C from 11 to 14 min. The conditions were held for 0.5 min before re-equilibrating the column at the starting conditions between 14.8 and 16 min.

The ionization of the compounds was performed using electrospray ionization in negative and positive modes, with polarity switching during the run. Selected Reaction Mode (SRM) was used for MS/MS acquisition. SRM transitions were individually optimized for targeted analytes and their respective internal standards using standard solutions. The isotopically-labeled internal standards used are detailed in **Table S2**.

For each target compound, the ratio of its peak area to the peak area of its corresponding internal standard was calculated using SCIEX OS-MQ Software and used for subsequent data analysis. Data quality was monitored through regular injections of quality control (QC) samples, which consisted of blank plasma samples, throughout the analytical sequence. QC samples were utilized to correct for inter-batch variations using the in-house developed mzQuality workflow (available at <http://www.mzQuality.nl>)[3]. Relative standard deviations (RSDs) of peak area ratios were calculated for each bile acid detected in the QC samples. The RSDs obtained for QC samples are listed in **Table S1.**

Total, primary, secondary, unconjugated, conjugated, glycine-conjugated, and taurine-conjugated bile acid peak area ratios were calculated as the sum of the corresponding individual species. In addition, we computed the secondary-to-primary, conjugated-to-unconjugated, and glycine-to-taurine conjugated bile acid ratios by dividing the respective summed values.

***Cardiometabolic risk factors***

Traditional cardiometabolic risk factors were determined in serum using standard procedures. Glucose levels were measured with an AU5832 analyzer (Beckman Coulter, Brea, CA, USA) using Beckman Coulter reagent (OSR6521). Insulin levels were assessed via chemiluminescence immunoassays on the UniCel DxI 800 analyzer (Beckman Coulter) with Beckman Coulter chemiluminescent reagents (33410). Total cholesterol (TC), high-density lipoprotein cholesterol (HDL-C), triglycerides (TG), apolipoproteins A and B, glutamic pyruvic transaminase (GPT), gamma-glutamyl transferase (GGT), alkaline phosphatase (ALP), creatinine and creatine kinase were measured using the AU5832 spectrophotometer with corresponding Beckman Coulter reagents (OSR6116, OSR60118, OSR6187, 446410, 447730, OSR6507, OSR6520, OSR6204 and OSR6678). Low-density lipoprotein cholesterol (LDL-C) was calculated using the Friedewald formula: [TC (mM) - HDL-c (mM) - 0.45 × TG (mM)]. C-reactive protein was assessed by immunoturbidimetric assays (OSR6299) using the AU5832 spectrophotometer. Leptin and adiponectin levels were measured in plasma using the MILLIPLEX MAG Human Adipokine Magnetic Bead Panel 2 (Catalogue # HADK2MAG-61K) and MILLIPLEX MAP Human Adipokine Magnetic Bead Panel 1 (Catalogue # HADK1MAG-61K), respectively, using a Luminex system (Luminex Corporation, Austin, TX, USA). Insulin sensitivity was estimated using the homeostatic model assessment of insulin resistance index (HOMA-IR) .

**Table S1.** List of bile acids measured.

| **Abbreviation** | **Common name** | **Name (International Union of Pure and Applied Chemistry, IUPAC)** | **ChEBI ID** | **RSD in QC** |
| --- | --- | --- | --- | --- |
| CA | Cholic acid | 3α,7α,12α-trihydroxy-5β-cholan-24-oic acid | 16359 | 26.8% |
| CDCA | Chenodeoxycholic acid | 3α,7α-Dihydroxy-5β-cholan-24-oic Acid | 16755 | 8.4% |
| GCA | Glycocholic acid | N-(3α,7α,12α-trihydroxy-5β-cholan-24-oyl)-glycine | 17687 | 10.8% |
| GCDCA | Glycochenodeoxycholic acid | N-(3α,7α-dihydroxy-5β-cholan-24-oyl)-glycine | 36274 | 8.4% |
| TCDCA | Taurochenodesoxycholic acid | N-(3α,7α-dihydroxy-5β-cholan-24-oyl)-taurine | 16525 | 20.8% |
| DCA | Deoxycholic acid | 3α,12α-Dihydroxy-5β-cholan-24-oic Acid | 28834 | 9.0% |
| UDCA | Ursodeoxycholic acid | 3α,7β-Dihydroxy-5β-cholan-24-oic Acid | 9907 | 12.5 |
| LCA | Lithocholic acid | 3α-Hydroxy-5β-cholan-24-oic Acid | 16325 | 18.7% |
| GDCA | Glycodeoxycholic acid | N-(3α,12α-dihydroxy-5β-cholan-24-oyl)glycine | 27471 | 8.8% |
| GUDCA | Glycoursodeoxycholic acid | N-(3α,7β-dihydroxy-5β-cholan-24-oyl)-glycine | 89929 | 13.8% |
| GLCA | Glycolithocholic Acid | N-[(3α,5β)-3-hydroxy-24-oxocholan-24-yl]-glycine | 37998 | 18.5% |
| TDCA | Taurodeoxycholic acid | N-(3α,12α-dihydroxy-5β-cholan-24-oyl)-taurine | 9410 | 16.5% |
| TUDCA | Tauroursodeoxycholic acid | N-(3-alpha,7-beta-Dihydroxy-5-beta-cholan-24-oyl) -Taurine | 80774 | 14.1% |
| TLCA | Taurolithocholic acid | N-(3α-hydroxy-5β-cholan-24-oyl)-taurine | 36259 | 12.9% |
| TLCA-3S | Taurolithocholic acid 3-sulfate | N-(3α-hydroxy-5β-cholan-24-oyl)-taurine 3-sulfate | 17864 | 12.9% |
| LCA-3S | Lithocholic acid 3-sulfate | 3α-sulfooxy-5β-cholan-24-oic acid | 35421 | 15.9% |
| HCA | Hyocholic acid | 3α,6α,7α-Trihydroxy-5β-cholan-24-oic acid | 81244 | 22.5% |
| THDCA | Taurohyodeoxycholic Acid | 2-[[(3α,5β,6α)-3,6-dihydroxy-24-oxocholan-24-yl]amino]-ethanesulfonic acid | 139138 | 13.3% |

ChEBI, Chemical Entities of Biological Interest; N/A, not available; ND, not detected; QC, quality control; RSD, relative standard error.

**Table S2.** List of metabolites with corresponding database identifiers.

| **Metabolite** | **Common_Name** | **HMDB_ID** | **Lipidmaps** |
| --- | --- | --- | --- |
| CA | Cholic acid | HMDB0000619 | LMST04010001 |
| CDCA | Chenodeoxycholic acid | HMDB0000518 | LMST04010032 |
| DCA | Deoxycholic acid | HMDB0000626 | LMST04010040 |
| DEA | Docosatetraenoyl Ethanolamide;Adrenoyl ethanolamide | HMDB0013626 | LMFA08040047 |
| GCA | Glycocholic acid | HMDB00138 | LMST05030001 |
| GCDCA | Glycochenodeoxycholic acid | HMDB0000637 | LMST05030008 |
| GDCA | Glycodeoxycholic acid | HMDB0000631 | LMST05030006 |
| GLCA | Glycolithocholic Acid | HMDB0000698 | LMST05030009 |
| GUDCA | Glycoursodeoxycholic acid | HMDB0000708 | LMST05030016 |
| HCA | Hyocholic acid | HMDB0000760 | LMST04010064 |
| HDCA | Hyodeoxycholic acid | HMDB0000733 | LMST04010024 |
| LCA | Lithocholic acid | HMDB0000761 | LMST04010003 |
| LCA-3S | Lithocholic acid 3-sulfate | HMDB0000907 | LMST05020015 |
| TCA | Taurocholic acid | HMDB0000036 | LMST05040001 |
| TCDCA | Taurochenodesoxycholic acid | HMDB0000951 | LMST05040005 |
| TDCA | Taurodeoxycholic acid | HMDB0000896 | LMST05040013 |
| THDCA | Taurohyodeoxycholic Acid |  |  |
| TLCA | Taurolithocholic acid | HMDB00722 | LMST05040003 |
| TLCA-3S | Taurolithocholic acid 3-sulfate | HMDB0002580 | LMST05020003 |
| TUDCA | Tauroursodeoxycholic acid | HMDB0000874 | LMST05040015 |
| UDCA | Ursodeoxycholic acid | HMDB0000946 | LMST04010033 |

**Table S3.** Sensitivity analysis of cold-induced changes in plasma bile acid concentrations after adjustment for PET-CT acquisition time.

|  | **All** | **NW** | **OW/OB** |
| --- | --- | --- | --- |
|  | **P value** | **P value** | **P value** |
| Total | **0.005** | **0.010** | 0.392 |
| Primary | 0.808 | 0.704 | 0.909 |
| Secondary | **0.007** | **0.004** | 0.343 |
| Unconjugated | 0.081 | **0.045** | 0.461 |
| Conjugated | 0.055 | **0.032** | 0.690 |
| Glycine-conjugated | 0.637 | 0.247 | 0.076 |
| Taurine-conjugated | 0.184 | 0.386 | 0.207 |
| Secondary/Primary | 0.414 | 0.356 | 0.957 |
| Conjugated/Unconjugated | 0.911 | 0.719 | 0.878 |
| Gly/Tau conjugated | 0.325 | 0.656 | **0.015** |
| CA | **0.030** | **<0.001** | **0.413** |
| CDCA | 0.683 | 0.591 | 0.882 |
| GCA | 0.102 | *0.065* | 0.985 |
| GCDCA | **0.260** | *0.056* | 0.734 |
| TCDCA | **0.110** | **<0.001** | **0.040** |
| DCA | 0.663 | 0.524 | 0.829 |
| UDCA | 0.805 | 0.935 | 0.504 |
| LCA | **0.135** | **0.043** | 0.774 |
| GDCA | 0.288 | 0.198 | 0.984 |
| GUDCA | 0.858 | 0.380 | 0.360 |
| GLCA | 0.512 | 0.109 | 0.057 |
| TDCA | **0.049** | **<0.001** | **0.035** |
| TUDCA | 0.477 | 0.493 | 0.929 |
| TLCA | 0.381 | 0.604 | 0.136 |
| TLCA-3S | 0.147 | 0.093 | 0.986 |
| LCA-3S | **0.048** | **0.022** | **0.663** |
| HCA | **0.018** | **0.012** | **0.348** |
| THDCA | 0.147 | **0.045** | 0.905 |

**
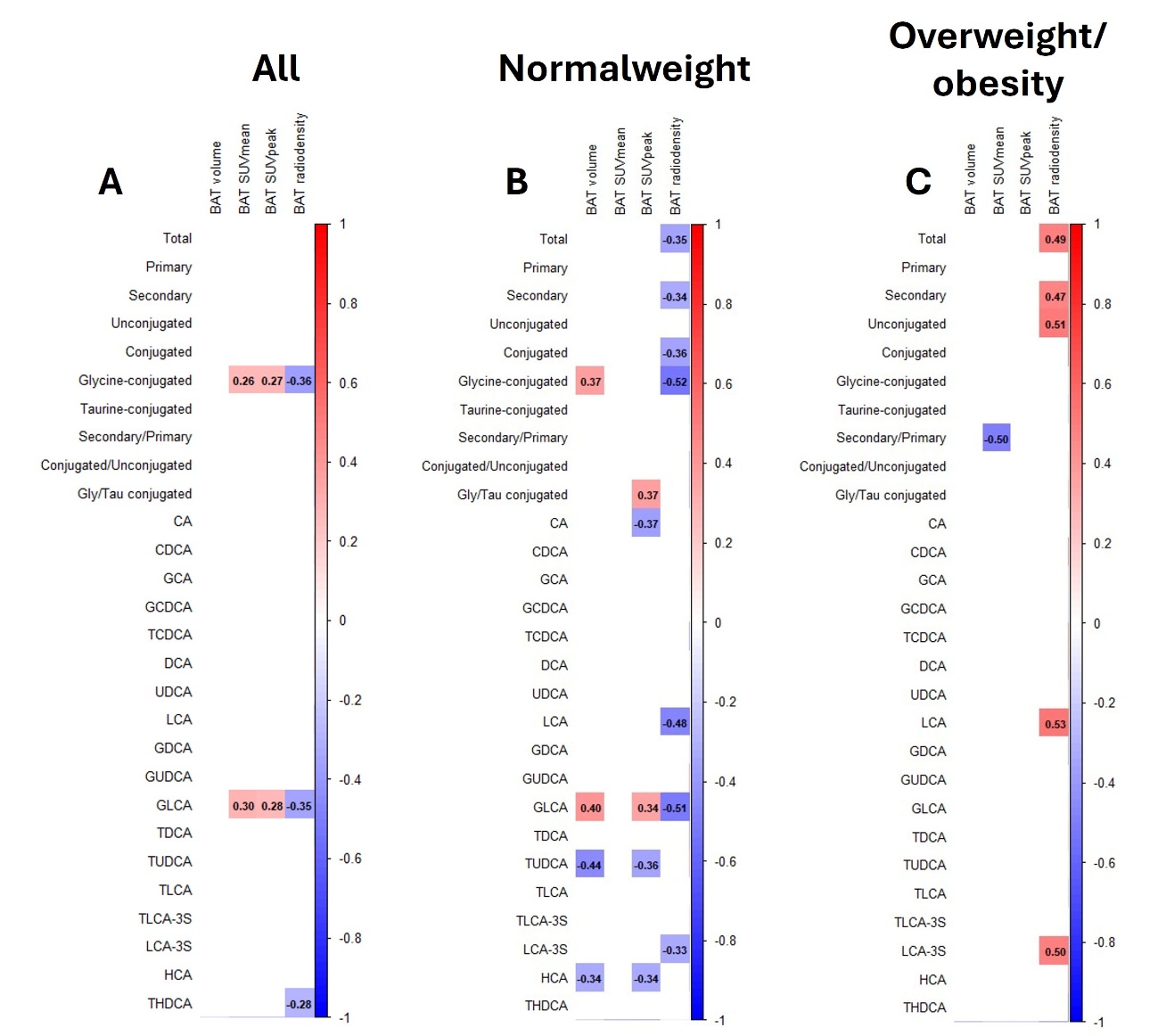
**

**Figure S1. Association between cold-induced changes in the plasma levels of bile acids and brown adipose tissue-related outcomes in all participants (A; n=56), normal-weight individuals (B; n=19), and individuals with overweight/obesity (C; n=37).** Pearson partial correlation analyses between the log2 120-min fold change relative to baseline and BAT-related outcomes adjusted for the natural calendar day and time when the baseline PET/CT scan was performed. Every box represents a significant correlation coefficient (all p<0.05 after FDR correction), whereas empty spaces represent no significant correlations. Red and blue boxes indicate positive and negative correlations, respectively. Abbreviations: BAT, brown adipose tissue; SUV, standardized uptake value. The names and abbreviations of bile acids are detailed in Table S1.

**
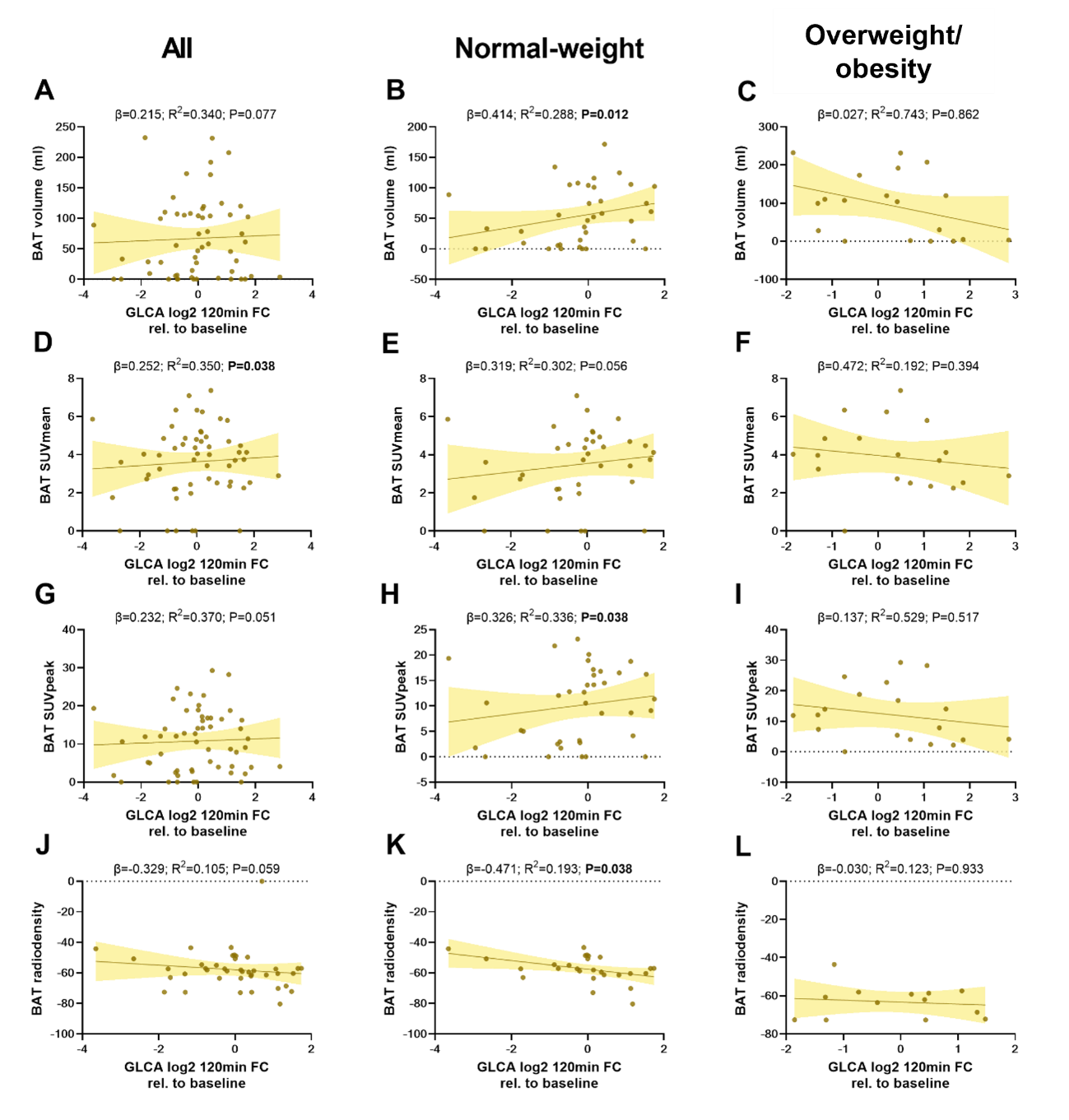
**

**Figure S2. Multiple linear regression analyses between glycolithocholic acid 120min log2 fold change rel. to baseline and BAT-related outcomes in all (A, D, G, J), normal-weight individuals (B, E, H, K), and individuals with overweight/obesity (C, F, I, L).** Each plot represents individual points, a regression line, and its corresponding 95% confidence interval. P-values were obtained from multiple linear regression analyses adjusted for the natural calendar day and time when the baseline PET/CT scan was performed. Bold P-values indicate statistically significant associations. Abbreviations: BAT, brown adipose tissue; FC, fold change; GLCA, glycolithocholic acid.


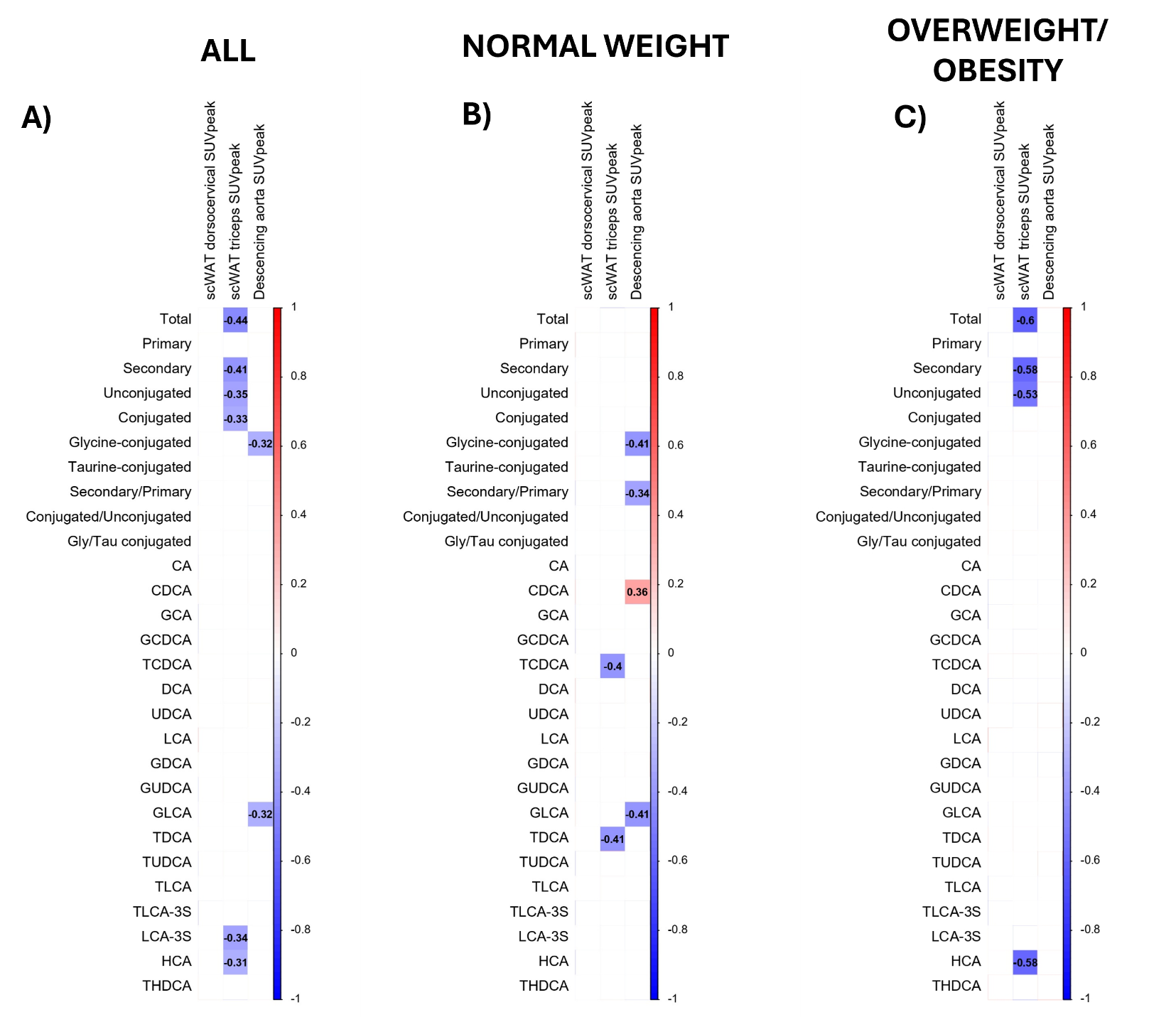


**Figure S3. Association between cold-induced changes in the plasma levels of bile acids with reference tissues in all participants (A; n=56), normal-weight individuals (B; n=19), and individuals with overweight/obesity (C; n=37).** Pearson correlation analyses between the log2 120-min fold change relative to baseline and reference tissues. Every box represents a significant correlation coefficient (all p<0.05 after FDR correction), whereas empty spaces represent no significant correlations. Red and blue boxes indicate positive and negative correlations, respectively. *Abbreviations*: scWAT, subcutaneous white adipose tissue; SUV, standardized uptake value**.** The names and abbreviations of bile acids are detailed in Table S1.


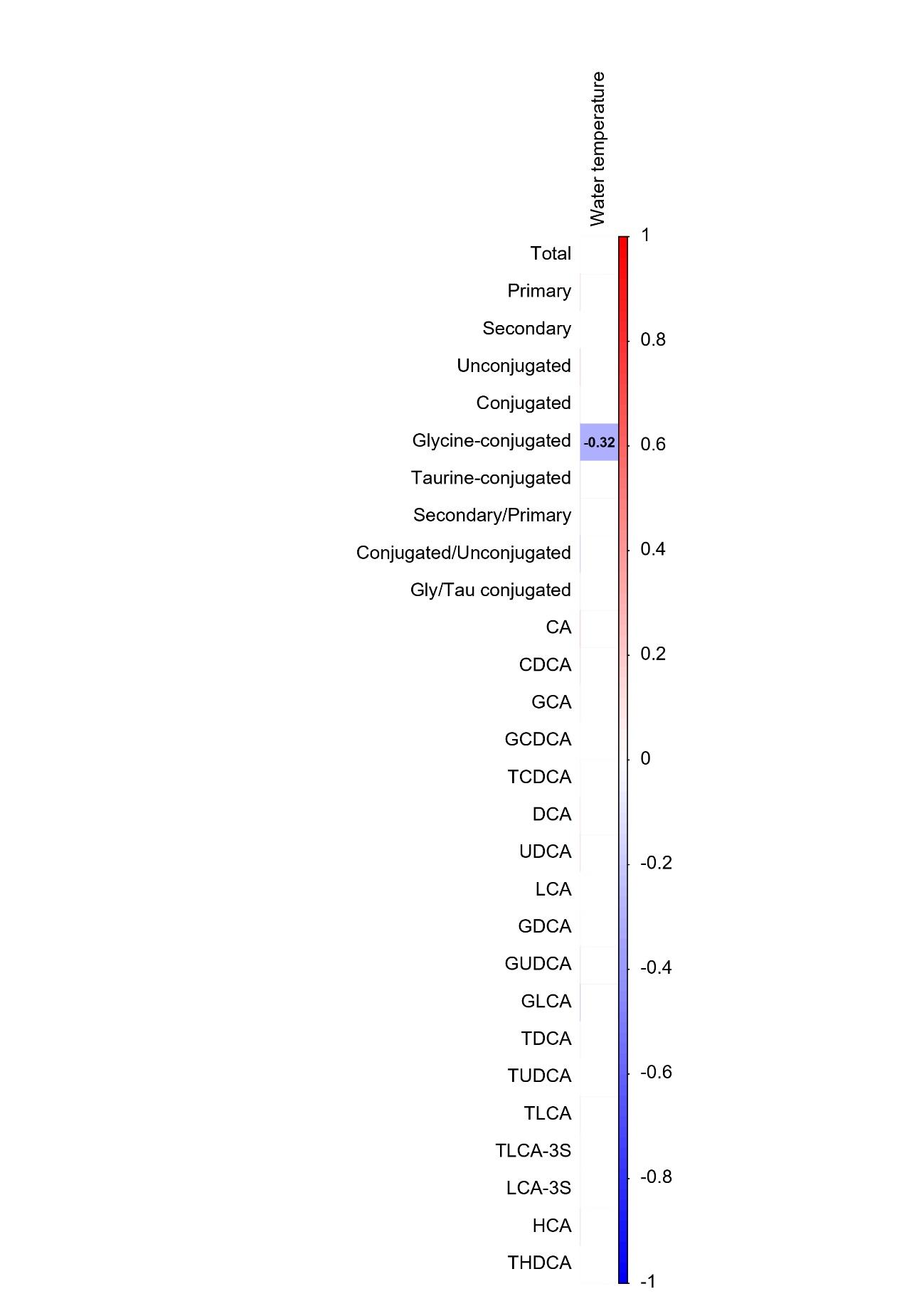


**Figure S4. Association between cold-induced changes in bile acids and the water temperature of the cooling vest.** Pearson correlation analyses between the log2 120-min fold change relative to baseline and water temperature of the cooling vest. Every box represents a significant correlation coefficient (all p<0.05 after FDR correction), whereas empty spaces represent no significant correlations. Red and blue boxes indicate positive and negative correlations, respectively. The names and abbreviations of bile acids are detailed in Table S1.

**REFERENCES**

1. Jurado-Fasoli L, Di X, Sanchez-Delgado G, et al (2022) Acute and long-term exercise differently modulate plasma levels of oxylipins, endocannabinoids, and their analogues in young sedentary adults: A sub-study and secondary analyses from the ACTIBATE randomized controlled-trial

2. Jurado-Fasoli L, Yang W, Kohler I, et al (2022) Effect of Different Exercise Training Modalities on Fasting Levels of Oxylipins and Endocannabinoids in Middle-Aged Sedentary Adults: A Randomized Controlled Trial. Int J Sport Nutr Exerc Metab 32:1–10. https://doi.org/10.1123/ijsnem.2021-0332

3. Van Der Kloet FM, Bobeldijk I, Verheij ER, Jellema RH (2009) Analytical error reduction using single point calibration for accurate and precise metabolomic phenotyping. J Proteome Res 8:5132–5141. https://doi.org/10.1021/pr900499r

4. Matthews JC (1985) Instability of brain synaptosomal membrane preparations to repeated ultracentrifugation in isoosmotic density gradients. Life Sci 37:2467–2473. https://doi.org/10.1017/CBO9781107415324.004
